# Mechanism of the Defect Formation in Supported Graphene by Energetic Heavy Ion Irradiation: the Substrate Effect

**DOI:** 10.1038/srep09935

**Published:** 2015-04-30

**Authors:** Weisen Li, Xinwei Wang, Xitong Zhang, Shijun Zhao, Huiling Duan, Jianming Xue

**Affiliations:** 1 State Key Laboratory of Nuclear Physics and Technology, School of Physics, Peking University, Beijing 100871, P. R. China; 2 Peking University Shenzhen Graduate School, Shenzhen 518055, P. R. China; 3 State Key Laboratory for Turbulence and Complex System, Department of Mechanics and Aerospace Engineering, College of Engineering, Peking University, Beijing 100871, P.R. China; 4 Center for Applied Physics and Technology, Peking University, Beijing 100871, P. R. China

## Abstract

Although ion beam technology has frequently been used for introducing defects in graphene, the associated key mechanism of the defect formation under ion irradiation is still largely unclear. We report a systematic study of the ion irradiation experiments on SiO_2_-supported graphene, and quantitatively compare the experimental results with molecular dynamic simulations. We find that the substrate is, in fact, of great importance in the defect formation process, as the defects in graphene are mostly generated through an indirect process by the sputtered atoms from the substrate.

Graphene has recently attracted great attention for a variety of novel applications, such as in nanoelectronics, gas detectors, solar cells, and DNA sequencing^1^,^2^,^3^,^4^,^5^. Engineering the graphene by ion beam technique is considered very promising, as it can easily realize the direct doping of foreign atoms^6^,^7^, and create defects in a controllable manner for further functionalization^8^,^9^. On the other hand, graphene is naturally very sensitive to form defects under ion irradiation^10^, but the key mechanism associated with this defect formation process is rather unclear, especially in the case of on a substrate as used in many experiments.

In ion-matter interaction theory, an energetic ion transfers its energy to the target system by both the elastic collisions with the target nuclei and the inelastic interactions with the target electrons (e.g. causing electronic excitations and/or ionization). In most of the previous reports^11^,^12^, the defect formation in graphene was completely attributed to the nuclear collisions, while the electronic excitations and ionization effects seem to be less important due to a high thermal and electrical conductivity of graphene, especially for the cases irradiated with keV ions^13^. However, recent work suggest that the effect of the electronic energy loss cannot be completely ignored in the defect formation process when the energy loss is extremely high, as for instance, swift heavy ions (91 MeV Xe) can even unzip the graphene due to its high electronic energy loss^14^.

On the other hand, the effect from the substrate underneath the graphene was also found playing an important role in the defect formation process. Compagnini et al.^12^ showed that, after 0.5 MeV C ion irradiation, the amount of the induced disorder was larger in a single layer graphene than in a multilayer graphene, and they attributed this difference to the stronger interaction of the single layer graphene with the SiO_2_ substrate. Contrarily, Mathew et al.^15^ suggested that the substrate supported graphene should be more resistive to the ion irradiation, as their measured damage threshold of substrate supported graphene under 2 MeV H ion irradiation was much higher than that of the suspended graphene. In addition, our simulations, based on molecular dynamics (MD), previously showed that the sputtered atoms from the substrate were very important in the defect formation process in graphene^16^. However, Buchowicz et al.^17^ claimed that the sputtered atoms from the SiO_2_ substrate under 35 keV C ion irradiation had negligible effect on the graphene. As described above, many apparently contradictory claims have been made, and the key mechanism of the defect formation under ion irradiation is still a mystery.

In this work, we report a systematic experimental study of the defect yield (*Y*) in graphene under energetic ion irradiation. A variety of ion species with a large range of the ion energy were used. We further performed MD simulations to compare the experimental results in order to understand the underlying mechanism. We found that the defect formation only depends on the nuclear collision until *S_e_* reaches a threshold of ~ 2.2 keV/nm, above which the electronic energy loss becomes important; moreover, the majority of the defects are actually created by the sputtered atoms from the substrate rather than the direct ion-graphene collisions. Therefore, the interaction between the energetic ions and the substrate should be paid great attention to, if one would like to use the ion beam technique for engineering graphene.

## Results

**Figure 1:**
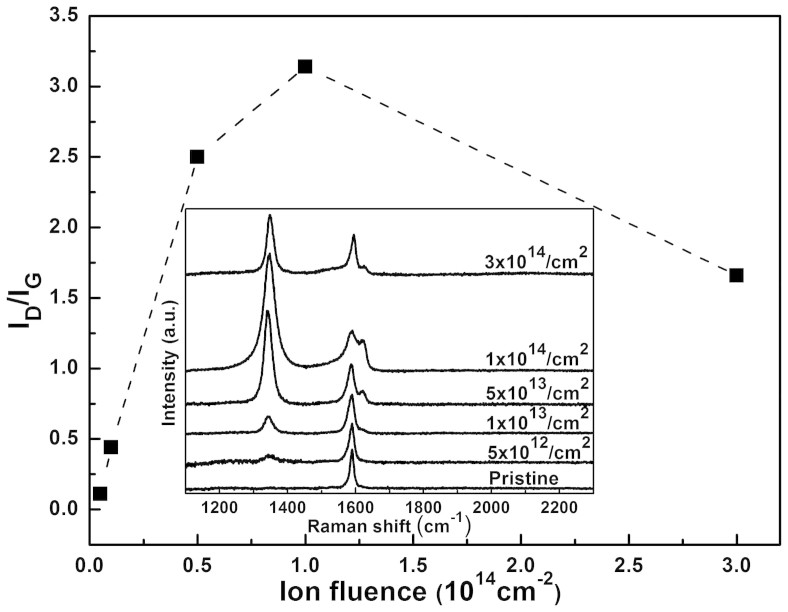
*I_D_/I_G_* ratio of the SiO_2_ supported graphene irradiated by 1 MeV C ions with different ion fluences. The insert shows the corresponding Raman spectra.

Figure 1 shows a typical Raman spectrum evolution of *I_D_/I_G_* ratio in SiO_2_-supported graphene after ion irradiation, where 1 MeV C ions were used. Similarly as was found previously^18^,^19^, the *I_D_/I_G_* ratio first increases linearly with the ion fluence, then gradually saturates and eventually decreases at high ion fluences, due to the defect coalescence effect. Based on the phenomenological model^19^,^20^, the defect yield can be directly extracted from the *I_D_/I_G_*ratio at low defect density, avoiding the defect coalescence effect (e.g. 5 × 10^12 ^ions/cm^2^ for the 1 MeV C case, as shown in Fig. 1). Therefore, the defect yields reported in the following were all obtained from the low-ion-fluence experimental results.

Our experimental results of the measured defect yields are listed in Table 1, where we also include some other experimental data adopted from literatures using other irradiation conditions (i.e., 250 MeV Sn^21^, 35 keV C^17^, 30 keV N^8^, and 100 keV Ar^22^). We also calculated, by using SRIM, the nuclear stopping power *S_n_*and the electronic stopping power *S_e_* of the incident ions in SiO_2_ and graphene (Table 1). For the graphene, the stopping values were adopted by using bulk graphite as the target in SRIM; even though the physical meaning of using keV/nm as the unit is less clear for graphene, we can still use these values to compare among different impacting ions, as the ion energy losses are correlated with the tendency/rate of transferring the energy away from the energetic impacting ions.

**Figure 2:**
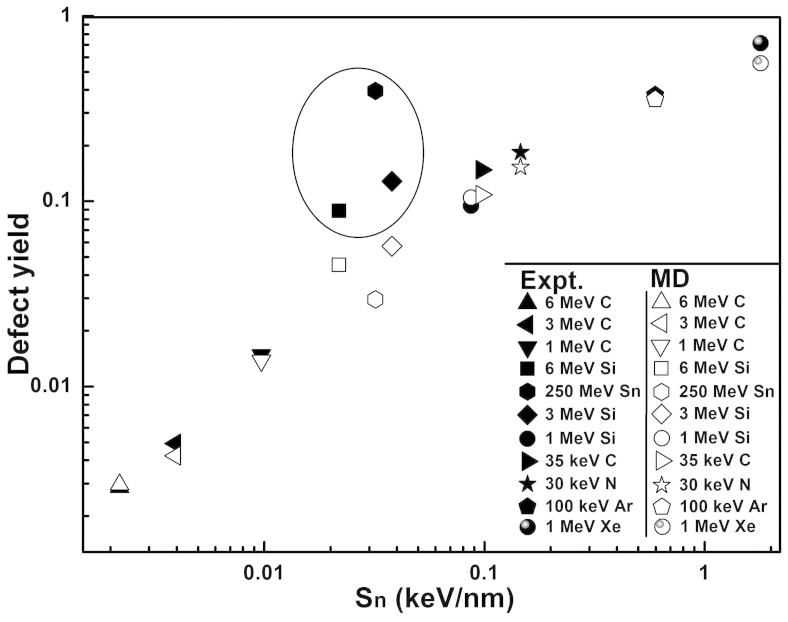
Experimentally measured (full symbols) and MD simulated (open symbols) defect yields in graphene (on SiO_2_ substrate) were plotted with respect to the nuclear stopping power (*S_n_*) of the irradiated ions in graphene. The ions of which the electronic energy loss cannot be ignored are circled in the figure and grouped as Group B, and the remaining ions are grouped as Group A (see text).

We first plot, as shown in Fig. 2, the experimental data (full symbols) of the defect yields against the nuclear energy stopping power of different impacting ions in graphene. In the same figure, the data from the MD simulations (open symbols) are also plotted for comparison. Surprisingly, the experimental data and the simulated data agree quantitatively well, except for the cases of 3, 6 MeV Si and 250 MeV Sn, where, as will be discussed later, the *S_e_*of these ions are so high that the electronic stopping process can no longer be ignored. For simplicity, we group these three exceptional ions as Group B and group the remaining ions as Group A.

## Discussion

For the ions in Group A, as shown in Fig. 2, the defect yield (*Y*) monotonously increases with *S_n_*, following almost a linear relation in the log-log plot. But no such positive correlation was found with respect to *S_e_*, although the electronic energy loss is in general the major energy loss pathway for MeV heavy ions (see Table 1). This is quite consistent with the previous studies^11^,^13^ that it is the nuclear collision process that dominates the defect formation in graphene. Our MD simulation, in which the electronic energy loss is neglected, further confirms this point, as the simulated defect yields match quantitatively well with the experimental data (Fig. 2). Note that, to make quantitative comparison with the experimental data, we adopt the phenomenological model^19^ to process our simulation results: each defect is circled with an activated region (activating the Raman D band) of 3 nm in radius, and the total area of the activated regions (not double counting the overlapped) is used to calculate the defect yield.

**Figure 3:**
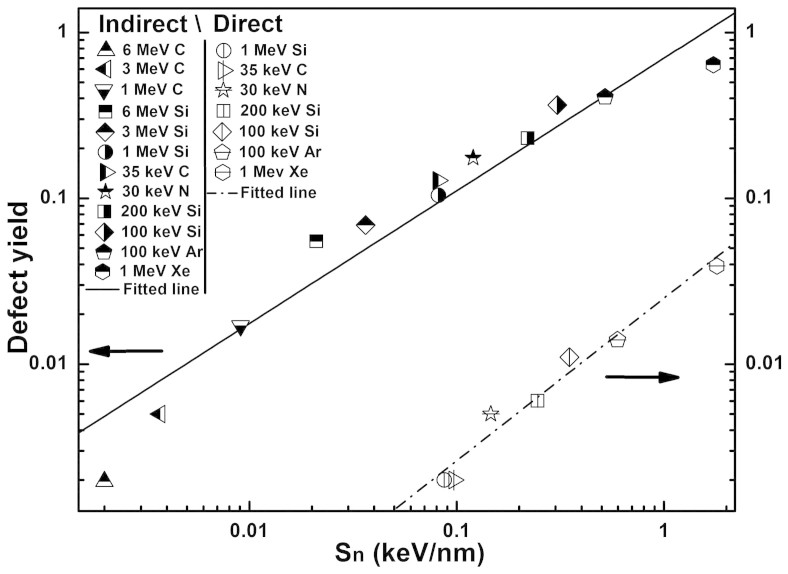
The defect yields from the direct (open symbols) and the indirect processes (half full symbols) were separately counted from the MD simulations, and are plotted with respect to *S_n_*in graphene and *S_n_*in SiO_2_, respectively. Power-law fitted curves are also plotted.

As for the nuclear collisions, one would expect that the defects are generated by direct collision process (direct process) where incident ions directly knock out the graphene carbon atoms and leave the vacancy-type defects in graphene. However, by carefully analyzing the simulation results, we found that the majority of the defect sites were laterally quite far (a few nanometers) from the incident spots of the impacting ions, thus they were unlikely generated by the direct process. In fact, they were generated by the sputtered atoms from the SiO_2_ substrate (indirect process). Nevertheless, the indirect process also includes the defects generated by the recoiled incident ions^16^; however, they are very rare as found in our simulation, so they are safely ignored in the present discussion. Note that, by this indirect process, the defects can be generated fairly far from the incident spots. Statistically, we choose the defect-to-incident-spot distance of 5 Å as the dividing point to separately count for the defect yields by the direct process (*Y_dir_*) and the indirect process (*Y_ind_*). As the results shown in Fig. 3, the indirect process is clearly dominating over the direct process for the entire range of *S_n_* used in this work: the defects generated by the direct process are consistently at least one order of magnitude less.

This dominating behavior can be understood in the following way. The displacement energy (*T_d_*) for a carbon atom to be knocked out from a freestanding graphene sheet is about 25 eV using the AIREBO potential^16^, which is slightly larger than 22.03 eV obtained in first-principles calculations^23^. This is because the AIREBO potential tends to overestimate the maximum force needed to break a carbon-carbon covalent bond^24^. However, for the graphene on SiO_2_, the displacement energy depends on the direction of the atoms being knocked out. We previously show that^16^ the displacement energy is much higher (more than 60 eV) for the carbon atoms to be knocked towards the SiO_2_ substrate, while it still remains around 25 eV for being knocked out into the vacuum. The former situation corresponds to the direct nuclear collision process with the impacting ions, thus the associated defect generation is greatly suppressed due to the high *T_d_*. The latter situation (towards vacuum) corresponds to the collisions initiated by the sputtered atoms from the substrate. Generally, the sputtered atoms carry energies spreading in a fairly wide range from several eV to several tens of eV following Thompson distribution^25^. With the SRIM simulation taking 1 MeV C ions impacting on bare SiO_2_ as an example (see Supplementary Fig. S1 online), as high as 20% of the sputtered atoms carry an energy higher than 25 eV. Thus, a large portion of the sputtered atoms can possibly knock out the graphene carbon atoms into the vacuum.

Based on the above analysis, we separately fit the direct and indirect defect yields by the power laws with respect to the nuclear stopping power in graphene (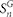) and in SiO_2_ (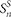), respectively, as the following:
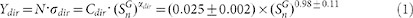

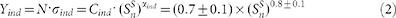
where *σ*_dir _and *σ*_ind _are the defect formation cross-sections for the direct and the indirect processes, respectively, N is the atomic density of carbon atoms in graphene, *C*_dir_ and *C*_ind_ are the fitted prefactors, and *α*_dir _and *α*_ind _are the fitted power exponents. The fitted lines are also plotted in Fig. 3. For the direct process, the fitted exponent (*α*_dir_) is fairly close to 1, so the defect formation cross-section follows a simple linear relationship with respect to the nuclear stopping power. This simple linear relationship suggests that the simple binary collision model can still be used to describe the defect formation by the direct process with only a slight modification on the displacement energy (resulting in the decreased prefactor). On the other hand, for the indirect process, the defect formation is rather complicated; the cross-section depends on many factors, such as the interaction between the impacting ions and the substrate, the sputtering yield of the substrate, the types of the sputtered species, and their kinetic energy distributions. Nevertheless, the defect yield still follows reasonably well with the power law of 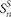, although small curvatures exist at high/low *S_n_*ends. The fitted parameters are practically useful, as they represent some universal features of the defect formation process, and can be used as a first-hand estimation for choosing the ion irradiation conditions in future.

For the ions in Group B, the experimental defect yields were clearly higher than the MD simulation results, as in which only nuclear collisions were considered. The experimental yields for 3 MeV and 6 MeV Si ions were roughly two times higher than the simulated counterparts, and the discrepancy became much larger when 250 MeV Sn ions were used (at least 10 times higher). This is because the electronic energy losses of these ions are so high (see Table 1) that the associated electronic energy transfer process can no longer be neglected for generating defects. However, graphene itself should be quite resistive to form defects by this electronic energy stopping process, because, according to the thermal spike model^26^, which is widely used to evaluate the damage from electronic energy loss, materials with higher thermal conductance usually have higher *S_e_*thresholds for defect forming, and graphene is known to have an extremely high thermal conductance of 5000 W/mK^27^. Even for the graphite, whose thermal conductance (2000 W/mK) is lower than graphene, still the reported experimental *S_e_* threshold is as high as 7.3 keV/nm^28^, which is much higher than the *S_e_* of 3 MeV Si (2.22 keV/nm) and 6 MeV Si (3.01 keV/nm) ions used in this work. On the other hand, we noticed that the substrate SiO_2_ has a considerably lower thermal conductance of only 1.44 W/mK^29^, thus the spiked thermal effect could accumulate more easily in SiO_2_. In fact, we previously showed that the sputtering yield of bare SiO_2 _started to increase when *S_e_* of the incident ions exceeds 2.2 keV/nm^30^. Thus, the increased defect formation was most likely to be formed by an indirect process in which the sputtering yield of SiO_2_ was enhanced by the high electronically lost energy of the incident ions. Accordingly, for this graphene-on-SiO_2_ system, the threshold of *S_e_* should be determined by the substrate, i.e., 2.2 keV/nm. In addition, it is worth mentioning that, via the strong electronic excitations, part of the sputtered particles might be ionized, like oxygen radicals, which are known to chemically react with graphene (e.g., graphene oxide etc.). As reported before^31^,^32^, chemical effect will also improve the defect production efficiency during collisions. To further corroborate the threshold, we performed the ion irradiation experiments on graphene using copper as the substrate. Copper is a good thermal conductor, so the effects from *S_e_*should be largely reduced. In fact, we found that, the experimental defect yield of graphene on copper was much reduced compared to that of SiO_2_ supported graphene (see Supplementary Fig. S2 online), both under the same irradiation conditions (6 MeV Si, 5 × 10^12^ ions/cm^2^).

**Figure 4:**
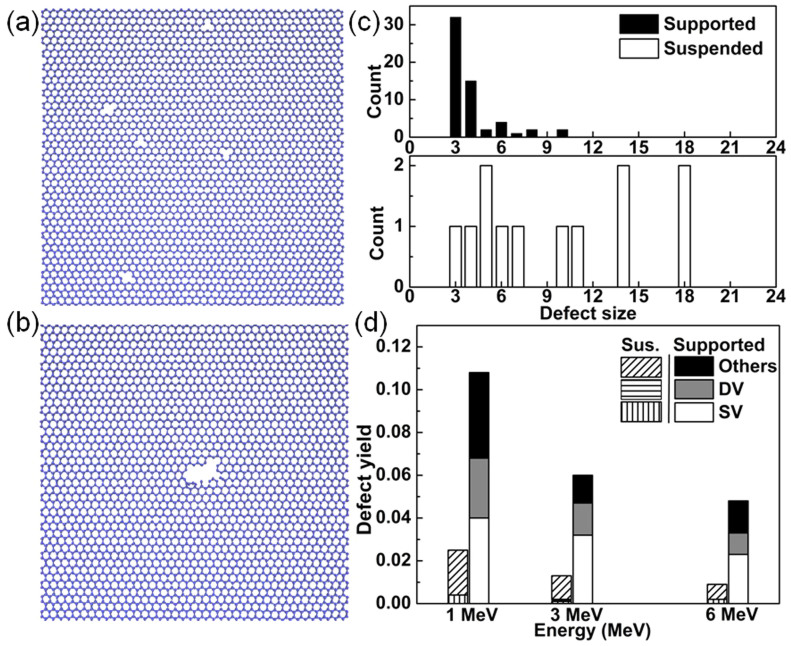
Typical MD-simulated defect patterns of (a) the SiO_2_-supported graphene and (b) the suspended graphene under 3 MeV Si ion irradiation. Small-size point defects (single vacancies (SV) or double vacancies (DV)) were preferentially formed in the SiO_2_-supported graphene, whereas large complex defects (pore) were the major defect format in the suspended graphene. The distributions of the defect size for (a) and (b) are compared in (c), respectively. Yields of different defect types in supported and suspended (Sus.) graphene sheets as a function of ion energy are compared in (d), where 1, 3, and 6 MeV Si ions are used for irradiations.

The defect patterns in the SiO_2_-supported graphene were further analyzed from the MD simulation results, and compared with the suspended graphene. Taking 3 MeV Si ion irradiation as an example, typical simulation snapshots are shown in Fig. 4a and Fig. 4b. We found that the defects with small size (single or double carbon vacancies) were preferentially formed in the SiO_2_-supported graphene, whereas, in the suspended graphene, large complex defects (pores) were the majority. Histograms comparing the size distributions of the defects are shown in Fig. 4c and Fig. 4d, where the defect size is defined as the number of the loss of the six-member carbon rings (e.g., defect size is 3 for single vacancy). In Fig. 4c, the difference is quite distinct: in the suspended graphene, more than 50% of the defects have the size greater than 10, while, in the supported graphene, this percentage is only around 5%, and the decrease of percentage for large defects in supported graphene mainly comes from an increased point defects production yield, as shown in Fig. 4d. For large complex defects, they are usually nanopore shaped, and were formed by fierce in-plane cascades^13^, which start with the impact of high energy ions and result in massive displacement of the graphene carbon atoms. On the other hand, for the supported graphene, the increased point defect production yields were mainly generated by the sputtered atoms from the substrate (indirect process). As the sputtered atoms usually carry relatively low energy, point defects were preferentially generated in the graphene^33^. This finding is practically very important, since it reveals a simple and effective way to improve the yield of small-size point defects in graphene. The point defects are usually considered as the active sites in graphene functionalization, thus post functionalization treatment, such as elemental doping8, and molecular adsorption^34^, should be easier for these ion irradiated samples.

## Conclusion

In summary, we have carefully investigated the mechanism of the ion-irradiation induced defect formation in the SiO_2_-supported graphene by systematic ion irradiation experiments and MD simulations. Our results clearly demonstrated the great importance of the substrate during the defect formation process. The direct nuclear collision process between the impacting ions and graphene carbon atoms can also generate defects, but in much less yield (at least one order of magnitude less) than the indirect process. Simple power-law fittings of the defect yields with respect to the nuclear energy losses were given for both the direct and the indirect processes, which can be, in future, used as a first-hand estimation for choosing the ion irradiation conditions. As for the electronic energy loss of the impacting ions, this portion of energy usually does not generate defects in graphene, unless the electronic energy loss of the ions is above a threshold of 2.2 keV/nm. We further reasoned that this threshold is actually set by the SiO_2_ substrate, as this value is the turning point for the sputtering yield of bare SiO_2_ to increase, which again high lights the importance of the substrate effect. In addition, our MD simulation also showed that the small-size point defects are preferentially formed in the supported graphene. Lastly, we believe that, by choosing the right substrate along with the right irradiation conditions, we can create defects in graphene with a highly controllable manner by the ion beam technique.

## Methods

### Sample preparation and disorder measurement

Samples of monolayer graphene on SiO_2 _substrate were used in the ion irradiation experiments. The monolayer graphene was grown by CVD on a copper foil, and was then transferred onto a SiO_2_(300nm)/Si substrate by standard transfer process^35^. After irradiation, Micro Raman spectroscopy with excitation radiation at 532 nm was used to measure the defect yield (*Y*) in graphene, i.e., the average number of the defect sites produced by one single incident ion. This defect yield was derived, based on a phenomenological model^19^,^20^, from the peak intensity ratio of the *D* band and the *G* band (*I_D_/I_G_*) in the Raman spectrum of the irradiated graphene. The *G* band (~1580 cm^−1^) is associated with the in-plane stretching motions of the sp^2^-bonded carbon atoms, whereas the *D* band (~1345 cm^−1^) corresponds to the defects in graphene^36^,^37^.

### Ion irradiations

C, Si ions irradiations were carried out on the 2 × 1.7 MV tandem accelerator at Peking university in Beijing, and Xe ions irradiations were carried out on the 320 kV ECR platform at IMP (Lanzhou). Ion energies, together with their stopping powers in SiO_2_ substrate and graphene, were listed in Table 1. All the irradiations were performed perpendicular to the graphene planes, at room temperature with ion fluences varied from 1 × 10^11^ to 3 × 10^14^ ions/cm^2^.

### MD simulation

For the simulation of the ion-solid interaction process, we used MD simulation to simulate this process in order to carefully investigate the mechanism of the defect formation in graphene under ion irradiation. In our MD simulation, the lateral size of the graphene/SiO_2_ slab (crystalline quartz) was 196.52 × 204.23 Å^2^, and the thickness was chosen to be around 60 Å. The adaptive intermolecular reactive empirical bond order (AIREBO) potential function^24^ was employed to calculate the interaction between carbon atoms. The Tersoff potentials with the parameters taken from Ref. 38 were applied to the substrate atoms (i.e., Si and O). The interaction between the graphene and the underlying SiO_2_ slab was assumed to be van der Waals (vdW) type, and was modeled by the Lennard-Jones potential with the parameters taken from Ref. 39. To model the energetic collisions, Ziegler-Biersack-Littmark repulsive potentials^40^ were used to describe the interaction between the incident ion and the substrate atoms (i.e., C, Si, and O) in the short distance. Electronic stopping process was not included in the simulation. The simulated defect yield was counted based on 1000 randomly selected impact events.

## Author Contributions

W.S.L. did the irradiations and Raman spectrum analysis of all the samples. W.S.L., X.T.Z., and S.J.Z did the MD simulations and defect statistics. W.S.L., X.W.W., H.L.D. and J.M.X. have written the paper. All authors have reviewed the manuscript.

## Supplementary Material

Supplementary Information Supporting information

## Figures and Tables

**Table 1:** Experimental defect yields (*Y*) in SiO_2_-supported graphene under various ion irradiation conditions used in this work. The yields were extracted based on the phenomenological model^19^,^20^ from the Raman spectra of the irradiated graphene samples. Also listed are the nuclear stopping power (*S_n_*) and electronic stopping power (*S_e_*) in SiO_2_ and graphene for each type of the impacting ions. All the stopping values were calculated by SRIM code

Ions	Defect yield (*Y*)	SiO_2_		Graphene	
		S_n_	S_e_	S_n_	S_e_
		(keV/nm)	(keV/nm)	(keV/nm)	(keV/nm)
6 MeV C	0.003	0.0021	1.31	0.0022	1.61
3 MeV C	0.005	0.0037	1.35	0.0039	1.64
1 MeV C	0.015	0.0092	0.98	0.0096	1.24
6 MeV Si	0.090	0.0213	3.01	0.0217	3.67
250 MeV Sn^21^	0.400†	0.0348	14.43	0.0340	16.29
3 MeV Si	0.130	0.0367	2.22	0.0378	2.76
1 MeV Si	0.096	0.0825	1.08	0.0870	1.49
35 keV C^17^	0.150†	0.0821	0.17	0.0970	0.32
30 keV N^8^	0.186†	0.1220	0.16	0.1500	0.33
100 keV Ar^22^	0.380†	0.5280	0.32	0.5900	0.62
1 MeV Xe	0.725	1.7300	0.75	1.8000	1.39

†These yield data were extracted from the literature Raman spectra.
