# Supplementary material for: Mechanism of the Defect Formation in Supported Graphene by Energetic Heavy Ion Irradiation: the Substrate Effect
Source: Sci Rep. 2015 Apr 30;5:9935. doi: 10.1038/srep09935 (PMC4415598; doi:10.1038/srep09935)
Supplement: Supplementary Information — Supporting information [file srep09935-s1.doc]

**Supporting Information**

Mechanism of the Defect Formation in Supported Graphene by Energetic Heavy Ion Irradiation: the Substrate Effect

Weisen Li,^1^ Xinwei Wang,^2, *^ Xitong Zhang,^1^ Shijun Zhao,^1^ Huiling Duan,^3^ and Jianming Xue^1,4, *^

*^1^State Key Laboratory of Nuclear Physics and Technology, School of Physics, Peking University,*

*Beijing 100871, P. R.China*

*^2^Peking University Shenzhen Graduate School, Shenzhen 518055, P. R. China*

*^3^State Key Laboratory for Turbulence and Complex System, Department of Mechanics and Aerospace Engineering, College of Engineering, Peking University, Beijing 100871, P.R. China*

*^4^ Center for Applied Physics and Technology, Peking University, Beijing 100871, P. R. China*

*Corresponding authors. E-mail addresses: [wangxw@pkusz.edu.cn](mailto:wangxw@pkusz.edu.cn) (Xinwei Wang), [jmxue@pku.edu.cn](mailto:jmxue@pku.edu.cn) (Jianming Xue)

**This file includes:**

- **Energy spectrum of sputter atoms from SiO_2_ substrate under 1 MeV C irradiation.**
- **Raman spectra of supported graphene on SiO_2_ and copper, under 6 MeV Si irradiation.**


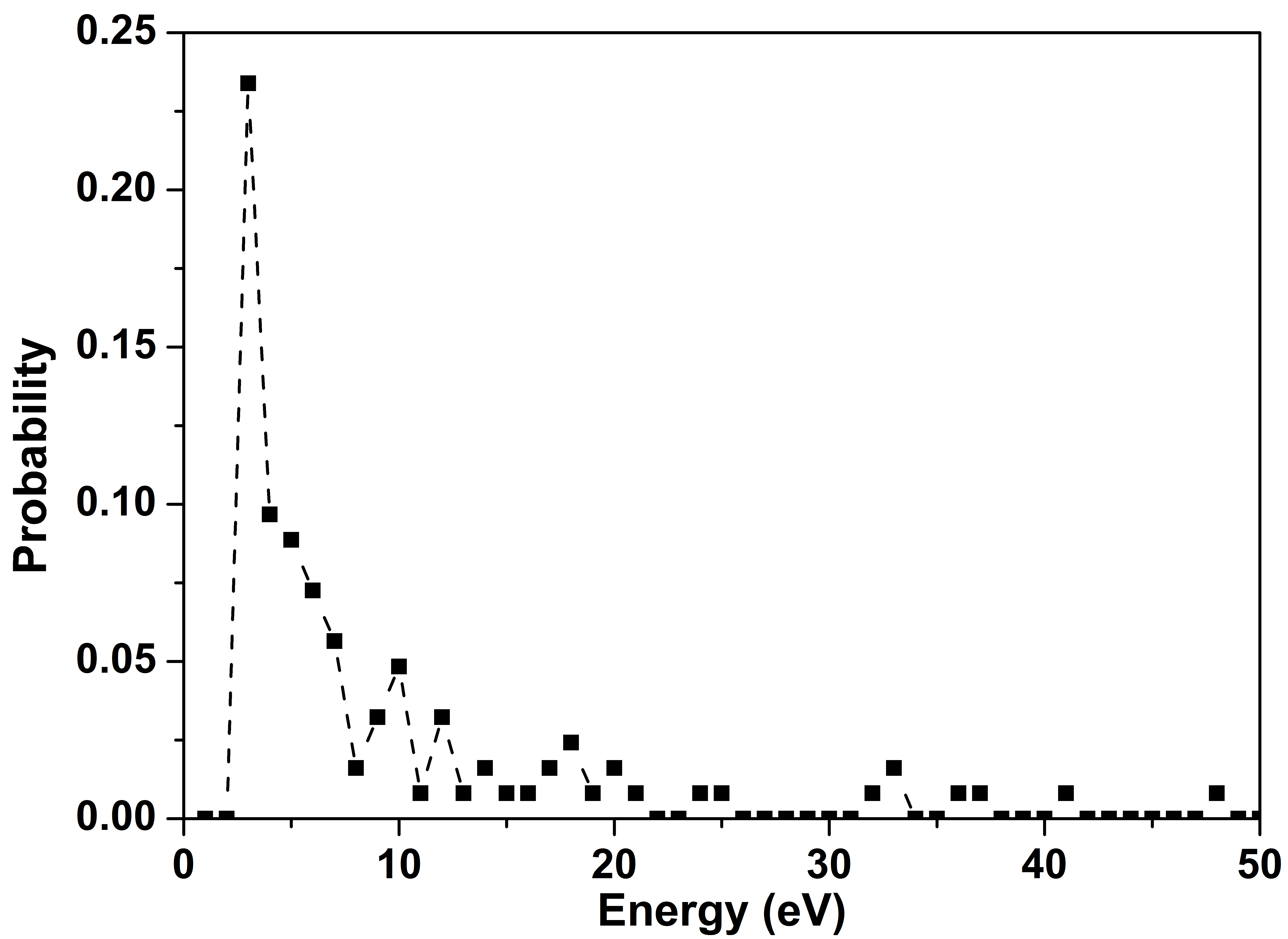


Fig. S1. Energy spectrum (< 50 eV) of the sputter atoms (i.e., O, Si) from SiO_2_ substrate under 1 MeV C irradiation, calculated by SRIM code. The majority of the sputtering atoms have rather low energies, with only about 20% proportion reach the displacement energy (T_d_ = 25 eV) of graphene carbon atom.


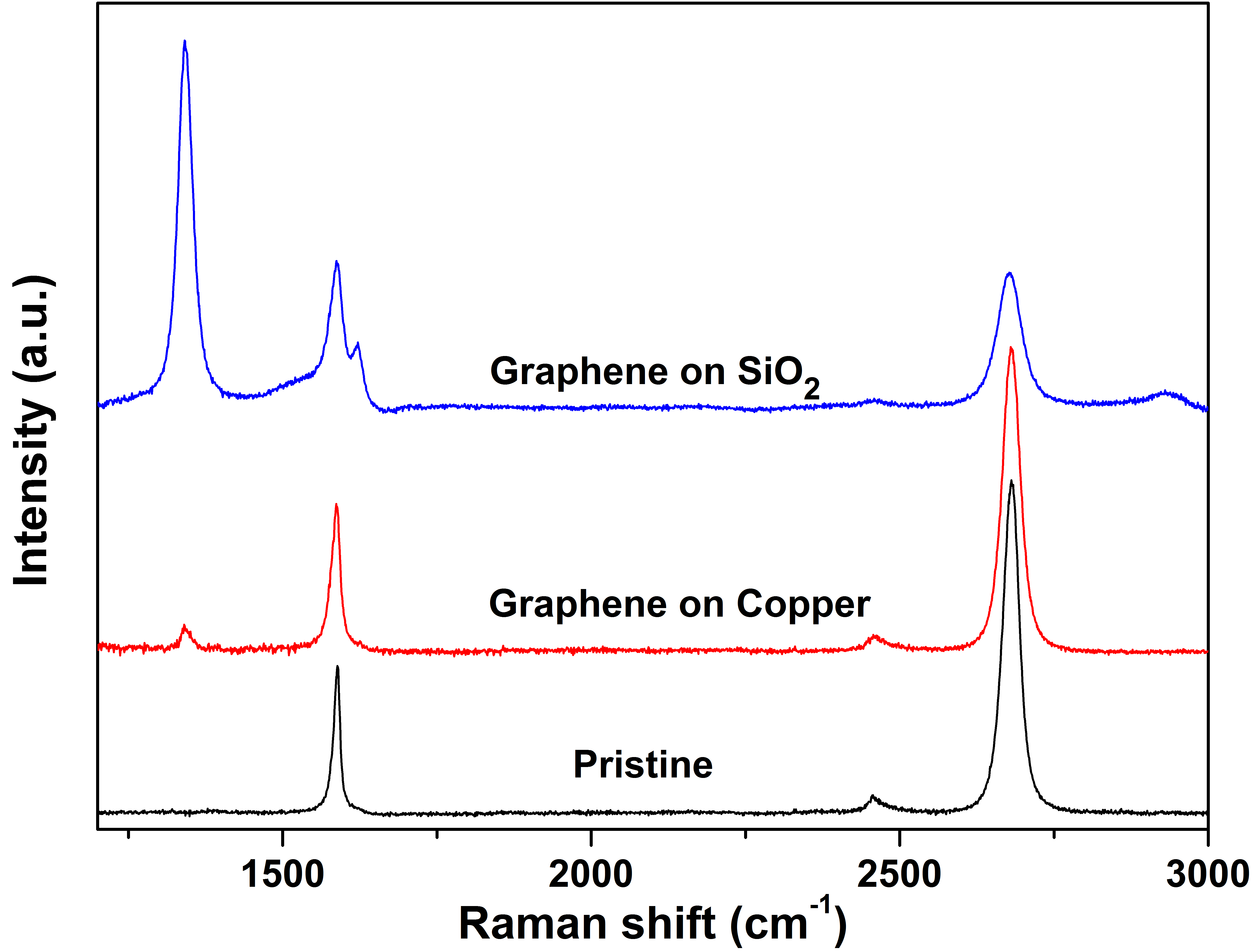


Fig. S2. Raman spectra of 6 MeV Si irradiated graphene on copper and SiO_2_(300nm)/Si substrates, respectively. The ion fluence was 5×10^12^/cm^2^. The large difference in graphene damage profile was attributed to be caused by different sputter yields in these two substrates.
